# A 5 year trend analysis of malaria prevalence with in the catchment areas of Felegehiwot referral Hospital, Bahir Dar city, northwest-Ethiopia: a retrospective study

**DOI:** 10.1186/s13104-017-2560-6

**Published:** 2017-07-04

**Authors:** Mulat Yimer, Tadesse Hailu, Wondemagegn Mulu, Bayeh Abera, Workneh Ayalew

**Affiliations:** 1 Department of Medical Microbiology, Immunology and Parasitology, College of Medicine and Health Sciences, Bahir Dar University, Bahir Dar, Ethiopia; 2 Amhara National Regional State Health Bureau, Felegehiwot referral Hospital, Bahir Dar, Ethiopia

**Keywords:** Malaria trend, *P. falciparum*, *P. vivax*, Ethiopia

## Abstract

**Background:**

Malaria is one of the killer diseases in Ethiopia and it is still the first leading cause of death in health facilities. However, there is no information yet regarding the trends of malaria prevalence at health institution and particularly at Felegehiwot referral Hospital. Hence , knowing the trends of malaria prevalence at each health facilities is essential to design appropriate interventions. Therefore, the present study addressed the above gaps.

**Results:**

Overall, 14,750 blood films were diagnosed for malaria. Of these, 740 (5%) were confirmed with microscope. *Plasmodium falciparum* and *Plasmodium vivax* accounted for 397 (53%) and 331 (45%), respectively. Age groups >20 year (p < 0.02) and males (p < 0.025) were significantly affected.

**Conclusions:**

In conclusion, *P. falciparum* was predominant as compared to *P. vivax*. Hence, it needs close monitoring and intervention measures for control activities.

## Background

In Ethiopia, according to the ministry of health report, *Plasmodium falciparum* (*P. falciparum*) accounts for 55% and *Plasmodium vivax (P. vivax*) for 45% of the cases. Reports also depicted that clinical malaria accounts for 10–40% of all out patient consultations and morbidity among children under 5 years in age being 10–20% [1].

In Ethiopia, four major intervention strategies are still applied to combat malaria [2]. Moreover, awareness creation and community mobilization were used as a main strategy for the control and prevention purposes. Despite these activities, malaria remains a major killer disease in the study area. Previously, chloroquine was the most commonly used antimalarial drug. However; increased in resistance to *P. falciparum* resulted in a change for treatment. As a result, artemether/lumefantrine (Coartem ^®^) (AL) was adopted [3, 4]. On the other hand, chloroquine is still the drug of choice for treatment of *P. vivax* even if, its resistance has not been yet known. Above all, self-treatment is common practice and this will result in development of drug resistance for the commonly prescribed drugs.

On top of these, different reports depicted confused results on decrement of malaria. Moreover, knowing the trends of malaria prevalence at each health institution is essential to design appropriate interventions. Furthermore, there is no information yet regarding the trends of malaria prevalence at health institution and particularly at Felegehiwot referral Hospital. Therefore, the present study addressed the above gaps.

## Methods

### Study area

A retrospective study was conducted to determine a 5 year trend analysis of malaria prevalence with in the catchment areas of Felegehiwot referral Hospital by reviewing malaria blood film malaria reports from 2010 to 2014. Bahir Dar city is situated on the Southern shore of Lake Tana, the source of the Blue Nile in what was previously the Gojjam province and now the Amhara National Regional State. The city is located approximately 578 km Northwest of Addis Ababa, having an elevation of 1840 meters above sea level. Based on the 2007 Census conducted by the Central Statistical Agency of Ethiopia, it has a total population of 221,991, of whom 108,456 are males and 113,535 females [5]. Since, Felegehiwot Hospital is a referral Hospital, malaria cases might come from malaria stable and unstable transmission areas.

### Data collection

5 year data on malaria cases were obtained from Felegehiwot referral Hospital. Laboratory technologists who examined blood films have 7–10 years of experience. To maintain the validity of this examination, a well-prepared and well-stained thin and thick blood films are used as the gold standard in confirming the presence of the malaria parasite as WHO protocol. The staining techniques and blood film examination for malaria parasite detection were conducted according to a standard operating procedure (SOP) stated on manual for the laboratory diagnosis of malaria [6] in each referral hospital throughout the country. Therefore, from July to August 2014, data on sex, age, type of *Plasmodium* species and season were collected on suspected malaria cases requested from 2010 to 2014 at Felegehiwot referral Hospital.

### Data analysis

Data was analysed using Statistical Package for Social Sciences (PSS) version 20. Descriptive statistics such as line and bar graphs were used to show the trends of malaria in terms of season and year, respectively. Chi-square test was used to compare the trends of malaria, male and female and also between different age groups. Finally, p < 0.05 was considered as statistical significant.

## Results

For the last 5 years (2010–2014), a total of 14,750 blood films were diagnosed for malaria at Felegehiwot referral Hospital. Of these, 7, 329 (49.7%) were males and 7, 421 (50.3%) were females with the median age of 23. The overall prevalence of microscopy—confirmed malaria parasitaemia was 740 (5%). Of these, *P. falciparum* accounted for 397 (2.7%) and *P. vivax* for 331 (2.2%). *Plasmodium* species infection had been observed more in males 406 (2.8%) than females 334 (2.3%). The difference was statistical significant (p < 0.025) (Table 1).

**Table 1:** Prevalence of malaria by sex at Felegehiwot referral Hospital for the last 5 years (2010–2014)

Sex	*Plasmodium* species					
	*P. falciparum* no (%)	*P. vivax* no (%)	Mixed no (%)*	Negative no (%)	Total no (%)	p value
Male	216 (2.9)	185 (2.5)	5 (0.07)	7087 (95.5)	7421 (50.3)	<0.025
Female	181 (2.5)	146 (2)	7 (0.1)	6923 (94.5)	7329 (49.7)	
Total	397 (2.7)	331 (2.2)	12 (0.08)	14,010 (95)	14,750 (100)	
p value	0.01					

* *Mixed infections* malaria cases positive for both *P. falciparum* and *P. vivax*

**Fig. 1:**
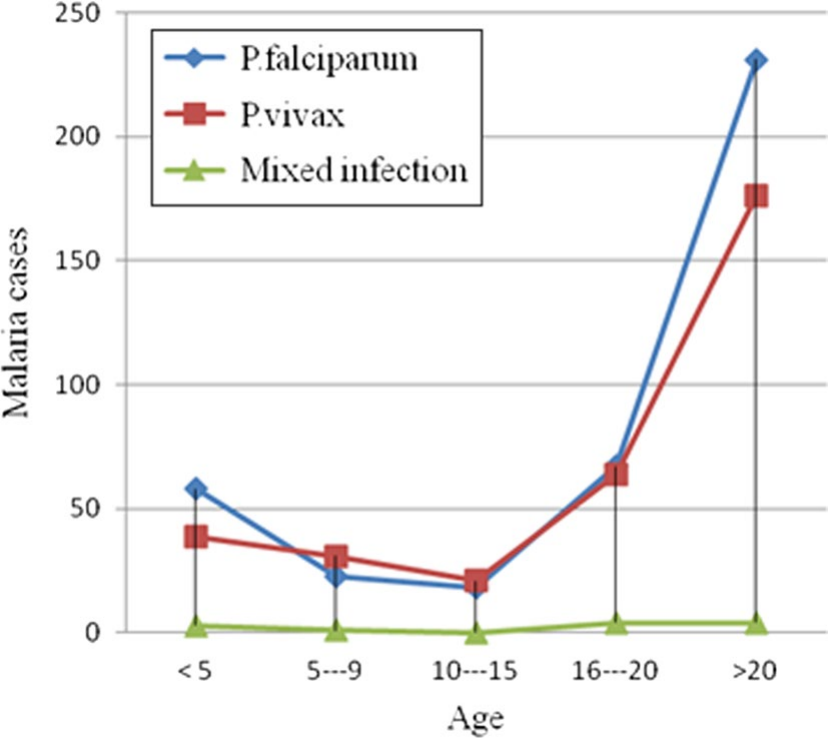
A 5 year trends of malaria by age group, 2010—2014

Trends of malaria by age group revealed that in <5 age groups, infection with *P. falciparum* was highest 58 (14.6%) as compared to *P. vivax* 39 (12%) and mixed infections 3 (2.5%). In age groups from 5 to 9 and 10 to 15, infection with *P. vivax* was dominant. In age groups from 16 to 20 and >20, there was a sharp increment in infection with both *P. falciparum* and *P. vivax*. Increments in mixed infections were observed from age groups 10–15 through >20 year. Statistical significant association was observed between age groups and infection with *P. falciparum* and *P. vivax* (p < 0.02) (Fig. 1).

**Fig. 2:**
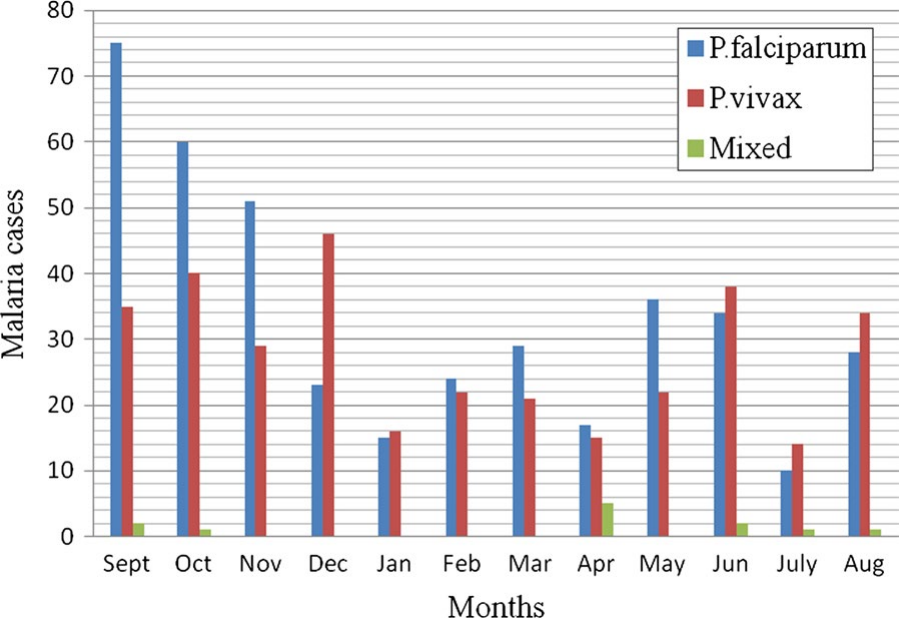
Monthly trends of malaria for a 5 year period at Felegehiwot referral Hospital from 2010—2014

A 5 year monthly trends of malaria showed that infection with *P. falciparum* was highest from September through November followed by February through May. More infection with *P. vivax* was observed from December to January followed by June through August. On the other hand, mixed infection was observed in September to October, in April and from June through August (Fig. 2).

**Fig. 3:**
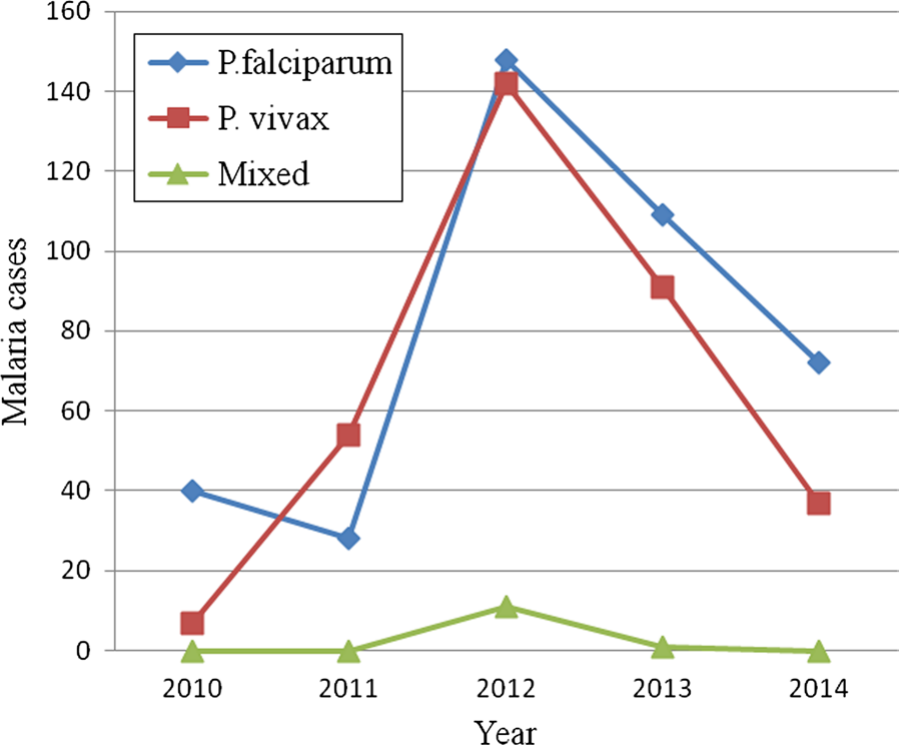
Species trends of malaria parasites at Felegehiwot referral Hospital from 2010—2014

Maximum cases with *P. falciparum*, *P. vivax* and mixed infections were observed in 2012 where as the least were observed in 2010. From 2011 to 2012, there was a sharp increment in *P. vivax* than *P. falciparum* infections. However, from 2012 through 2014, fast decrement in *P. vivax* than *P. falciparum* infections were observed. Finally, from 2011 to 2012, there was increment in mixed infections and from 2012 to 2013 there was decrement (Fig. 3).

## Discussion

The present study revealed that microscopy- confirmed malaria in 5 year at Felegehiwot referral Hospital was 740 (5%). This finding was smaller than a retrospective study done in Northwest Ethiopia [6] and Southwest Ethiopia [7–9]. This difference might be due to study period and geographical difference. It was also smaller than the National Ministry of Health report [1]. This might be due to data on Ministry of Health is reports from via out the country while our report is from the study area. Moreover, there might be inter-personal variation on malaria microscopists and this might affect the result.

In this study, the overall prevalence of malaria in males 406 (2.8%) was higher than females 334 (2.3%). This result was in line with the study done in Southwest Ethiopia [8]. This might be due to the fact that agriculture is the main job and sleeping and staying outdoor is common during the night time. And hence, males are more exposed to *Anopheles* mosquito bites.

The prevalence of *P. falciparum* 397 (2.7%) and *P. vivax* 331 (2.2%) in the present study was lower than studies reported from other parts of the country [8, 10]. This difference might be due to climatological differences and altitude variation.

In our study, the age groups, >20 years 411 (55.5%) were highly affected followed by 16–20 years old 135 (18.2%) but from 10 to 15 years old 39 (5.2%) were the least affected. This study coincides with a study done in Northwest [7], but different from a report in Southwest Ethiopia [9]. The possible reason might be due to responsibility of these age groups for caring of the family and hence, the probabilities of staying outdoor for a longer period.

Months and seasonality have a direct role in the transmission of malaria. In our study, the prevalence of *P. falciparum* throughout the year revealed that it seems stable transmission. The reason might be since Felegehiwot Hospital is a referral Hospital; new cases could be referred from malaria stable transmission areas. The highest prevalence was observed from September to December followed by May to June. This result was in agreement with the monthly trends of malaria transmission stated in ministry of health [11]. This monthly occurrence indicates the real malaria transmission.

According to this study, there were irregular occurrences of cases each year for the last 5 years. From 2011 to 2012, more *P. vivax* infection was observed compared to *P. falciparum* with the peak at 2012 which seems epidemic. This was in line with a report in Southwest Ethiopia [9]. This might be due to an overlooked feature of *P. vivax* [11, 12]. The limitation of this study was that we used microscopy having lower sensitivity rather than polymerase chain reaction (PCR) to identify *Plasmodium* species in the study area.

## Conclusions

The 5 year prevalence of malaria in our study area was lower than the National Ministry of Health. Therefore, more work will be done using PCR and RDT tests having more sensitivity.
